# Mesenchymal Stem Cells Genetically Modified by Lentivirus-Express Soluble TRAIL and Interleukin-12 Inhibit Growth and Reduced Metastasis-Relate Changes in Lymphoma Mice Model

**DOI:** 10.3390/biomedicines11020595

**Published:** 2023-02-17

**Authors:** Adriana G. Quiroz-Reyes, Carlos A. Gonzalez-Villarreal, Alberto Y. Limon-Flores, Paulina Delgado-Gonzalez, Herminia G. Martinez-Rodriguez, Salvador L. Said-Fernandez, Adolfo Soto-Dominguez, Ana M. Rivas-Estilla, Jose F. Islas, Juan F. Molina-De la Garza, Elsa N. Garza-Treviño

**Affiliations:** 1 Department of Biochemistry and Molecular Medicine, School of Medicine, Autonomous University of Nuevo Leon (UANL), Monterrey 64460, Mexico; 2 Department of Basic Sciences, Laboratory of Molecular Genetics, University of Monterrey (UDEM), Monterrey 66238, Mexico; 3 Department and Service of Immunology, School of Medicine, Autonomous University of Nuevo Leon (UANL), San Nicolás de los Garza 64460, Mexico; 4 Department of Histology, School of Medicine, Autonomous University of Nuevo Leon (UANL), San Nicolás de los Garza 64460, Mexico

**Keywords:** mesenchymal stem cells 1, lymphoma 2, TNF-related apoptosis-inducing ligand 3, interleulin-12 4, sTRAIL 5

## Abstract

Background: Cancer treatment has many side effects; therefore, more efficient treatments are needed. Mesenchymal stem cells (MSC) have immunoregulatory properties, tumor site migration and can be genetically modified. Some proteins, such as soluble TRAIL (sTRAIL) and interleukin-12 (IL-12), have shown antitumoral potential, thus its combination in solid tumors could increase their activity. Materials and Methods: Lentiviral transduction of bone marrow MSC with green fluorescent protein (GFP) and transgenes (sTRAIL and IL-12) was confirmed by fluorescence microscopy and Western blot. Soluble TRAIL levels were quantified by ELISA. Lymphoma L5178Y cells express a reporter gene (GFP/mCherry), and TRAIL receptor (DR5). Results: An in vivo model showed that combined treatment with MSC expressing sTRAIL+IL-12 or IL-12 alone significantly reduced tumor volume and increased survival in BALB/c mice (*p* < 0.05) with only one application. However, at the histological level, only MSC expressing IL-12 reduced tumor cell infiltration significantly in the right gastrocnemius compared with the control group (*p* < 0.05). It presented less tissue dysplasia confirmed by fluorescence and hematoxylin–eosin dye; nevertheless, treatment not inhibited hepatic metastasis. Conclusions: MSC expressing IL-12, is or combination with BM-MSC expressing sTRAIL represents an antitumor strategy for lymphoma tumors since they increase survival and reduce tumor development. However, the combination did not show significative additive effect. The localized application did not inhibit metastasis but reduced morphological alterations of tissue associated with liver metastasis.

## 1. Introduction

Despite different treatments, cancer is a leading cause of morbidity and mortality worldwide [1]. Therefore, it is necessary to continue developing new and better treatment strategies [2]. Tumor necrosis factor-related apoptosis-inducing ligand (TRAIL), a type II transmembrane protein, is a potent and specific proapoptotic protein ligand that activates the extrinsic apoptosis pathway of cell death receptors [3]. TRAIL in its bounding membrane form has been reported as more efficient than soluble TRAIL (sTRAIL) in activating apoptosis in target cells. However, in other studies, sTRAIL successfully induced apoptosis [4,5,6]. Recombinant TRAIL comprises a non-covalently assembled homotrimer that induces apoptosis selectively in tumor or transformed cells but not in normal cells. However, it may pose several limitations as a therapeutic agent for routine clinical use because of the pharmacologic instability of systemically delivered proteins and problematic distribution kinetics. Interleukin 12 (IL-12) is a pleiotropic cytokine that plays an essential role in the Th1-type immune response against cancer [7]. Unfortunately, its antitumor efficacy in preclinical models has not been replicated in humans, and it is extremely toxic [8].

New delivery strategies are necessary to facilitate their continuous administration in low doses. Mesenchymal stem cells (MSCs) transduced with genes expressing antitumor products have promising applications. In addition to being relatively easy to obtain and culture, they can migrate and release antitumor products directly in malignant tumor cells. This study evaluated the antitumor efficacy of implanting bone marrow (BM)-MSCs transduced with sTRAIL and/or IL-12 transgenes in a syngeneic lymphoma model in BALB/c mice. 

## 2. Materials and Methods

### 2.1. Isolation and Characterization of MSC

BM-MSC were isolated from 6 to 8-week-old BALB/c mice bone marrow from our institutional laboratory research animals. Mice were sacrificed in a CO_2_ chamber. Then, their femurs were decontaminated with 70% ethanol per minute. After that, the epiphysis was cut with a no. 10 scalpel. Bone marrow was extracted by introducing a syringe with a 30-caliber needle into the medullar channel. The syringe contained 2–4 mL of DMEM/F12-GlutaMAX (Gibco, Life Technologies, Grand Island, NY, USA) supplemented with 10% fetal bovine serum (FBS) (Gibco, Life Technologies, Grand Island, NY, USA), gentamicin (100 μg/mL) (Gibco, Life Technologies, Grand Island, NY, USA), amphotericin B (2.5 μg/mL) (Gibco, Life Technologies, Grand Island, NY, USA), and glutamine (2 nm) (Gibco, Life Technologies, Grand Island, NY, USA). The injected medium was further collected and deposited in a 25 cm^2^ culture flask (Corning Inc., Corning, NY, USA). Adherent cells were expanded an additional three passes in supplemented DMEM/F12-glutaMAX [6,9]. The following immunohistochemistry markers were used to identify MSC, CD105, CD90, and CD34, with the primary monoclonal antibodies anti-CD90, anti-CD105, and anti-CD34 (United States Biologicals, Salem, MA, USA), and PBS diluted 1:25, 1:200, and 1:100, respectively, as the ISCT specifies [10]. A mouse- and rabbit-specific HPR/DAB (ABC) detection IHC kit (Abcam, Burlingame, CA, USA) and Harris hematoxylin as a counterstain were used. 

### 2.2. Evaluation of BM-MSC Multipotency

The Mouse Mesenchymal Stem Cell Functional Identification Kit (R&D Systems, Inc. Minneapolis, MN, USA) was used for multipotency evaluation of BM-MSC to differentiate osteoblasts and adipocytes. First, adipocytes were dyed with oil O red to demonstrate lipid droplets. Osteocytes were dyed with Von Kossa to demonstrate bone matrix deposits. BM-MSC culture without differentiation was used as the negative control. Adipocytes and macerated rat bone were positive controls for oil O red and Von Kossa, respectively. 

### 2.3. Lentiviral Transduction of BM-MSC

The lentivirus was previously designed by our group [6] with the vector building platform by Cyagen (Santa Clara, CA, USA) for expression of sTRAIL and IL-12: pLV[Exp]-EGFP/Neo-EF1A > {sMurTRAIL}, pLV[Exp]-EGFP/Neo-EF1A > {MurIL12-70p}. Additionally, the lentiviral vector pLV[Exp]-EGFP:T2A:Puro-EF1A > mCherry was purchased in Vector Builder. It provides GFP green and mCherry red fluorescence. Transduction was performed following the methodology by Yuan et al. [11]. Culture flasks were seeded with 5 × 10^5^ BM-MSC and incubated for 16 h with 5% CO_2_ at 37 °C. Protamine sulfate 5 μg/mL (Sigma-Aldrich, Merck, St. Louis, MO, USA) was added after 16 h. All cultures were infected with lentivirus at 2 MOI (multiplicity of infection). Next, cells were incubated for an additional 48 h under the same conditions. Then, geneticin (200 μg/mL) (Gibco, Life Technologies, Grand Island, NY, USA) was added. Transduction efficiency was evaluated by fluorescent count using epifluorescence microscopy (Nokia, Eclipse 50i).

### 2.4. Transgene Expression Validation

Western blot was used for transgene expression assurance. After 48 h of culture, 9 ± 1 mL of culture medium was collected from flasks with transduced cells of sTRAIL and IL-12 genes. Next, the culture medium was concentrated by ultrafiltration and centrifugation in an Amicon^®^ Ultra-15 10K Centrifugal Filter Devices column (Merck-Millipore, Burlington, MA, USA) at 5000 RPM for 40 min at room temperature. Proteins were quantified with Bradford reactive Bradford Quick Start (Bio-Rad, Hercules, CA, USA). A 96-well plate in ice was used for protein stabilization. One μL per well of the sample was added along with 100 μL of Bradford reactive. Next, the reaction was incubated for five minutes on ice, followed by an absorbance read at 600 nm. Polyacrylamide gel electrophoresis (12%) was run with these preparations. Gels were loaded with 40 μL (approx. 100 μg/mL of total protein) and ran for 10 min at 80 V and then for 1 h at 100 V. Proteins were then transferred to a PVDF membrane (Bio-Rad, Hercules, CA, USA), and blocked overnight with silk milk 5% (Svelty, Nestlé, Mexico City, Mexico) in TBS in agitation (20 RPM) at 4 °C. A PVDF membrane was then incubated with mouse primary antibodies anti-TRAIL (1:200), and anti-IL-12 (1:1000) obtained from rabbit (Abcam). Anti-rabbit secondary polyclonal antibody conjugated with HRP (hose-radish peroxidase, 1:10,000) was also used. The Luminol Clarity ECL Kit (Bio-Rad, Hercules, CA, USA) was used to reveal. 

### 2.5. Quantification of Soluble TRAIL

Soluble TRAIL levels were quantified by ELISA using the TRAIL ELISA kit from Abcam (ab253210). sTRAIL-MSC 48 h culture supernatants were collected and centrifuged at 2000× *g* for 10 min. After that, ELISA was developed following the manufacturer’s instructions. A Cytation 3 (BIOTEK, Winooski, VT, USA) reader was used for TRAIL determination. 

### 2.6. Lymphoma Cell Gene Modification

Lymphoma tumors were produced in BALB/c mice using the murine lymphoma cell line L5178Y TK+/− clone (3.7.2C) [TK+/−(clone 3.7.2C] (ATCC^®^ CRL-9518™) (ATCC, Manassas, VA, USA). The cell line was cultured in a 25 cm^2^ culture flask (Corning, Tewksbury, MA, USA) with high glucose DMEM 5 mL supplemented with 10% FBS, gentamicin (50 μg/mL) (Gibco, Life Technologies, Grand Island, NY 1072, USA), amphotericin B (2.5 μg/mL) (Gibco, Life Technologies, Grand Island, NY 1072, USA) and Pluronic F68 (0.1%) (Gibco, Life Technologies, Grand Island, NY 1072, USA). Additionally, the lymphoma cell line was transduced with lentivirus pLV[Exp]-EGFP:T2A:Puro-EF1A > mCherry following the same methodology as BM-MSC to have red and green fluorescent cells. 

### 2.7. Metastasis Analysis

Two groups of 8- to 12-week-old female BALB/c mice were inoculated with L5178Y cells to standardize and analyze metastasis; one group was inoculated with 5 × 10^5^ GFP/mCherry transduced L5178Y cells in 100 μL saline solution. The second group was inoculated with 5 × 10^5^ L5178Y non-transduced cells in 100 μL saline solution, both in the right gastrocnemius. Mice were kept alive until day 22 when the tumor represented 10% of the mice’s total weight. They were then sacrificed in a CO_2_ chamber (70%) with posterior cervical dislocation. After the mice were sacrificed, a tumor biopsy was performed, and the following organs were isolated: liver, lungs, kidneys, and brain. The extracted tissues were embedded in Tissue-TEK O.C.T. compound (Sakura Finetek, Nagano, Japan), and 6 μm tissue sections were made in a cryostat (Roundfin, Shenyang, China). One drop (7 μL) of Vectashield with DAPI mounting medium (Vecta Mount, Vector laboratories, Burlingame, CA, USA) was added to tissue slices. They were then observed with an epifluorescence microscope (Nokia, Eclipse 50i), allowing an analysis of tumor cell distribution and metastasis. Histological analysis was completed with a hematoxylin–eosin (HE) stain. Immunohistochemical analysis of TRAIL receptor (TRAIL R2/DR5) (anti-DR5, Abcam, Burlingame, CA, USA) was conducted in 6 μm tumor slices from both groups to evaluate TRAIL apoptosis.

### 2.8. Experimental Treatments

Groups of five BALB/c 8- to 12-week-old female mice were formed and inoculated with 5 × 10^5^ GFP/mCherry L5178Y transduced cells in 100 μL saline solution into the right gastrocnemius to establish solid tumors. The weight and size of the leg were measured every third day during the murine model; however, the last measurement was made the day the mice died or were sacrificed. Each treatment consisted of 1 × 10^6^ BM-MSC suspended in 100 μL of saline solution directly in the tumor with the following combinations: group one, 100 μL of saline as a control; group two, 1 × 10^6^ naïve BM-MSC; group three, 1 × 10^6^ BM-MSC-IL-12; group four, 5 × 10^5^ BM-MSC-sTRAIL and 5 × 10^5^ BM-MSC-IL-12. Treatments were inoculated in mice when the tumor reached a size of 1–2 cm^3^.

### 2.9. Survival Time Determination

Tumors were followed until day 50 or whenever euthanized. Mice with the following signs were sacrificed: low ears, tense eyes, bristly hair, inactivity, and poor reaction to stimuli [12,13]. Additionally, if a mouse showed a decrement of 20% of its initial weight, it was euthanized. 

### 2.10. Post-Implant Tumor Evolution and Metastasis Analysis

Mice were individually analyzed lengthwise, registering deaths, collecting tumor biopsies, and mice weight, and based on findings from a previous experiment, infiltrated organs were collected. Extracted tissues embedded in Tissue-Tek O.C.T compound (Sakura Finetek, Nagano, Japan) were stored at −80 °C. Tumors were placed in cold acetone for 10 min as a pre-fix process and later embedded in Tissue-Tek O.C.T compound. Two sections of 6 μm were obtained with a cryostat (Roundfin, Shenyang, China) from tumor biopsies. They were then mounted on a slide and stained with H&E. Slides were observed by light microscopy, starting with fixed organs. Three sections of 6 μm per tissue were taken, and an H&E stain was performed to identify histologic changes characteristic of metastasis. We used established tumor cell infiltration parameters to develop a semiquantitative scale: 0, none; 1, scarce; 2, moderate; 3, abundant. Morphology was staged as follows: 1, without dysplasia (Non-SD); 2, mild dysplasia (Mild-DS); 3, moderate dysplasia (Moderate-DS); 4, severe dysplasia (Severe-DS). We screened 8 fields per slice, per triplicate, and analyzed them by chi-square. We evaluated lymphoma cells L5178Y and BM-MSC by epifluorescence.

### 2.11. Statistical Analysis

Mice survival was analyzed with the Kaplan–Meier test; tumor cell infiltration was analyzed by chi-square. We used one-factor ANOVA and the Kruskal–Wallis test for group comparison. The results were interpreted with SPSS v.22, GraphPad Prisma 9 software, and the Mantel–Cox log rank (significance, *p* ≥ 0.05).

### 2.12. Ethics

This study was approved by the Ethics in Research Committee and the Institutional Animal Care and Use Committee (IACUC) with registration number BI19-00003. The animals were handled according to current international regulations and the Official Mexican Norm for handling experimentation animals (NOM-062-ZOO-1999).

## 3. Results

### 3.1. Genetic Modified BM-MSC Are Capable of sTRAIL and IL-12 Overexpression

**Figure 1:**
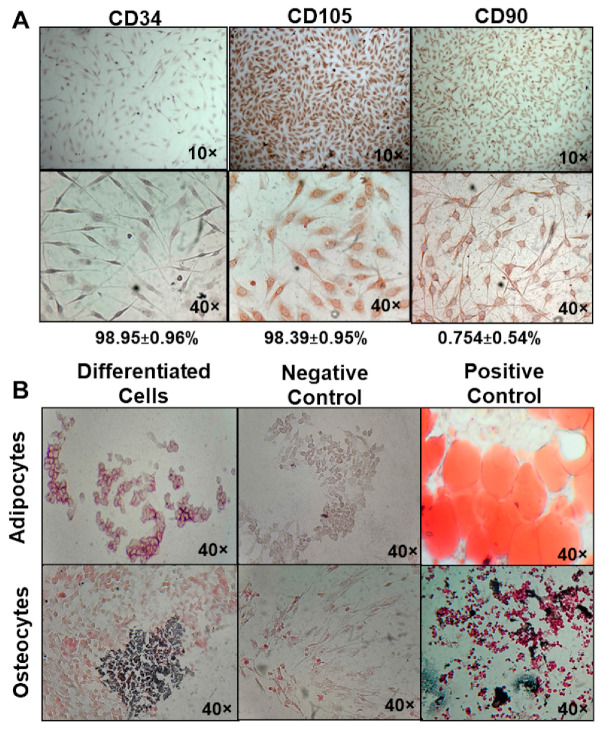
Characterization of BM-MSC from mice. (**A**) Antibodies anti-CD90 (1:200), anti-CD105 (1:25), and anti-CD34 (1:100). The percentage of positive cells in five fields. Positive, brown–red coloration; negative, blue–purple by hematoxylin counterstain. (**B**) BM MSC differentiated into adipocytes and osteocytes. First line: adipogenic linage differentiation: oil red O stain; positive red color in intracellular lipid drops, 14 days post-differentiation. Negative control: MSC without differentiation medium; positive control: rat adipose tissue. Second line: osteogenic lineage differentiation; positive as a dark brown precipitate of bone matrix, 21 days post-differentiation. Negative control: MSC without differentiation medium; positive control: rat bone. Bright field, 40×.

BM-MSC adherent cells derived from BALB/c mice were cultured for two weeks and stained for mesenchymal markers. Immunohistochemistry showed that bone marrow adherent cells had CD105^+^ (98.39 ± 0.945%), CD90^+^ (98.95 ± 0.964%), and CD34^−^ (0.754 ± 0.541%) phenotypes, corresponding to bone marrow mesenchymal stem cells (BM-MSC). Positive cells were identified by brown–red coloration, and negatives were observed as blue–purple due to Harris hematoxylin counterstain (Figure 1A).

BM-MSC can differentiate into different cell lineages, so we induced adipogenic and osteogenic differentiation. Adipocyte-derived BM-MSC changed their morphology to round cells, presenting intracellular lipid droplets and a slightly dyed red color. Osteoblast-derived BM-MSC presented a cuboid morphology and showed dark brown bone matrix deposits around cells (Figure 1B). 

**Figure 2:**
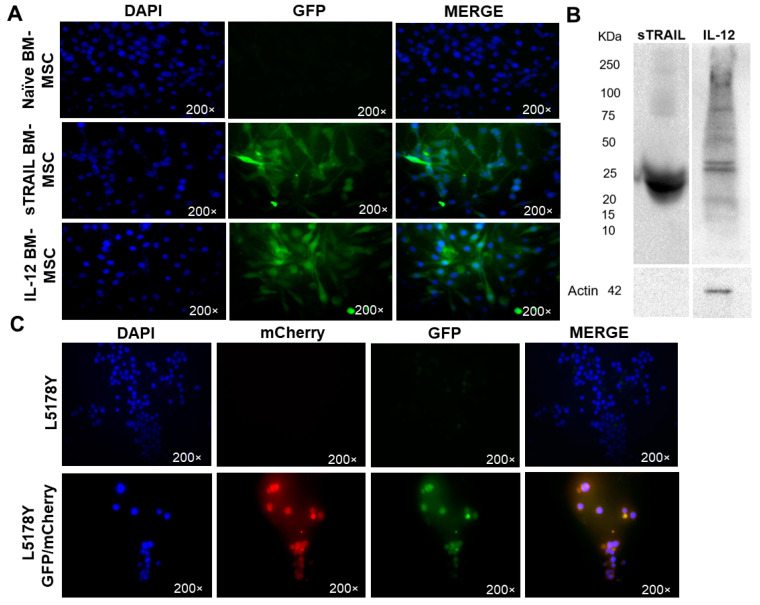
Transduced cells expressing proteins. (**A**) BM-MSC marked with GFP in green, the cell nucleus stained with DAPI in blue. Transduction efficiency: 88% sTRAIL MSC and 77% IL-12. MSC, mesenchymal stem cells; sTRAIL, soluble tumoral necrosis factor-related apoptosis-inducing ligand; IL-12, interleukin 12. Epifluorescence, 200×. (**B**) Western blot bands of BM-MSC expressed transgenes, sTRAIL, and IL-12. Sample volume 40 μL, 80–100 μg/mL of total protein. Actin control. Antibodies: anti-TRAIL (1:200) and anti-IL-12 (1:1000), anti-actin (1:1000), and HRP conjugated rabbit secondary antibody (1:10,000). Protein correspondent bands are black. Molecular weight: sTRAIL, 22 KDa; IL-12, 25 KDa; Actin, 42 KDa. (**C**) Lymphoma L5178Y cells marked with fluorescent reporter gen mCherry and GPF in red and green. Cell nucleus stained with DAPI in blue. Transduction efficiency: 94%. Epifluorescence, 200×.

GFP expression efficiency by transduced BM-MSC was determined by counting fluorescent cells using epifluorescence microscopy. Gene expression validation was analyzed by Western blot. Transduced BM-MSC expressed GFP as a tracking marker. We randomly selected four fields of transduced BM-MSC at 40×, showing 88% sTRAIL BM-MSC and 77% IL-12 BM-MSC GFP-positive cells (Figure 2A). Western blot assured gene expression at the protein level. Transduced BM-MSC-corresponding bands of sTRAIL and IL-12 proteins were detected in conditioned media. Figure 2B shows the results. 

Plus, we quantified soluble TRAIL levels in supernatants. Protein levels by ELISA had a mean of 328.3 ± 90.13 pg/mL of sTRAIL produced by transduced MSC at 48 h.

### 3.2. L5178Y Lymphoma Cell Line Develops Liver Metastasis

**Figure 3:**
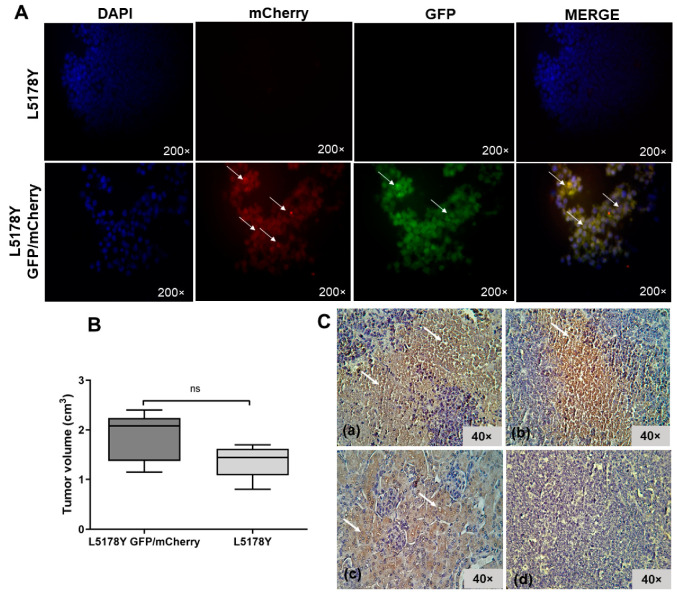
Transduced L5178Y cells in tumors. (**A**) Fluorescent detection of transduced L5178Y cells in the tumor. Comparison of transduced and non-transduced lymphoma L5178Y cell line. White arrows indicate a transduced cell marked with the reporter gene GFP and mCherry in green and red. Cell nucleus stained with DAPI in blue. Epifluorescence, 200×. (**B**) Tumor volume at day 9. Graphic values represent media ± standard error. ns: no significant difference, *p* > 0.05. (**C**) Murine TRAIL (DR5) receptor in tumor samples at day 22 post-inoculation. (**a**) Group inoculated with non-transduced L5178Y cells, 60% positivity; (**b**) group inoculated with transduced L5178Y cells, 60% positivity; (**c**) positive control (mouse kidney) and (**d**) negative control (tumor without primary antibody). Positivity indicated by brown–red coloration; cell nucleus in purple by hematoxylin counterstain. Analysis of 10 random fields, n = 3. Slices of 6 μm in bright field at 40×.

**Figure 4:**
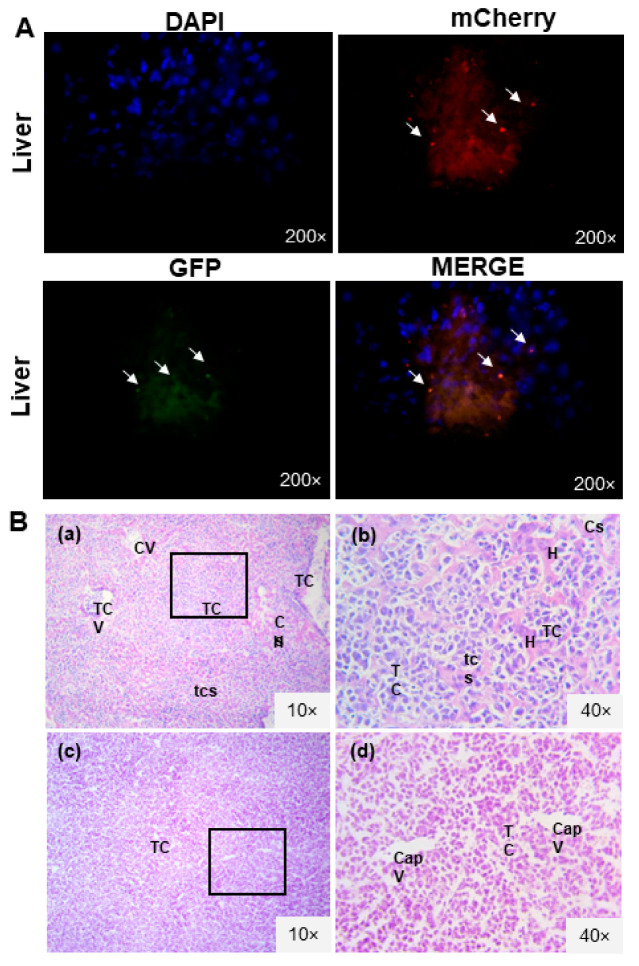
Liver metastasis analysis. (**A**) Fluorescence images of the brain, lungs, liver, and kidney. Positive tissues to transduced L5178Y cells presented the reporter gene mCherry signal in red and GFP in green (white arrows). Cell nucleus stained with DAPI in blue. Epifluorescence, 200×. (**B**). Analysis by hematoxylin–eosin. (**a**) Liver cut from the inoculated group with transduced L5178Y cells, bright field 10×. Metastasis is evidenced by purple tumoral cells in the central vein, sinusoids, and hepatic parenchyma as tissue dysplasia. Hepatocytes in pink. (**b**) Metastatic liver 40× amplification. Tumoral cell invasion is observed in sinusoids. TCV: Central vein with tumoral cells. CV: Central vein with blood cells. TC: tumoral cells. H: Hepatocytes. Sv: Clean sinusoids. Sct: Sinusoids with tumoral cells. (**c**) Tumor slice of group inoculated with transduced L5178Y cells, which was used as a positive control of tumoral cells, bright field 10×. Absence of muscle cells proper of skeletal muscle tissue. (**d**) A 40× amplification of tumor; tumoral capillaries are observed. TC: tumoral cells. Vcap: capillary vessels in the tumor.

Lentiviral transduction of a lymphoma cell line was developed like BM-MSC. Genetic modification with reporters allowed tumor cell identification in tissues through fluorescence (GFP/mCherry). The transduction process did not modify the normal morphologic characteristics of tumor cells since an intact membrane was observed (Figure 2C). There was also no significant difference in tumor volume between the two mice groups on the ninth day (*t*-test, *p* > 0.05). This finding showed that the transduction procedure did not alter the cell line ability of solid tumor development (Figure 3B). The fluorescent signal from transduced GFP/mCherry L5178Y lymphoma cells (Figure 3A) was confirmed in tumors. Next, the liver, lungs, brain, and kidneys were analyzed. In mice from the group inoculated with transduced lymphoma cell line, a fluorescent signal from mCherry and GFP in liver sections was detected as a red and green color, respectively, indicating possible liver metastasis (Figure 4A). Tumor cell damage was confirmed by microscopic analysis of each tissue. Tumoral lymphoid cells in sinusoids around hepatocytes, new blood vessel formation, and hepatic parenchyma dysplasia were found (Figure 4B). There was no fluorescent signal in other isolated tissues (kidneys, lungs, and brain). Morphologic changes corresponding to metastasis were not observed. 

### 3.3. The L5178Y Lymphoma Cell Line Expresses TRAIL Receptor 

TRAIL receptor presence was analyzed in tumor slices from both groups inoculated with L5178Y transduced and non-transduced cells. First, we sacrificed animals on day 22. In both groups, we demonstrated that cells were positive for the DR5 receptor (Non-transduced = 60%, transduced = 60%). Positive cells were observed with brown–red cytoplasmic coloration, indicating that the cells are sensitive to apoptosis by the TRAIL ligand. However, the DR5 signal was not continuous and occurred in separate areas (Figure 3C). 

### 3.4. IL-12 MSC and the Combination plus sTRAIL MSC Reduce Tumor Growth and Improve Mice Survival

**Figure 5:**
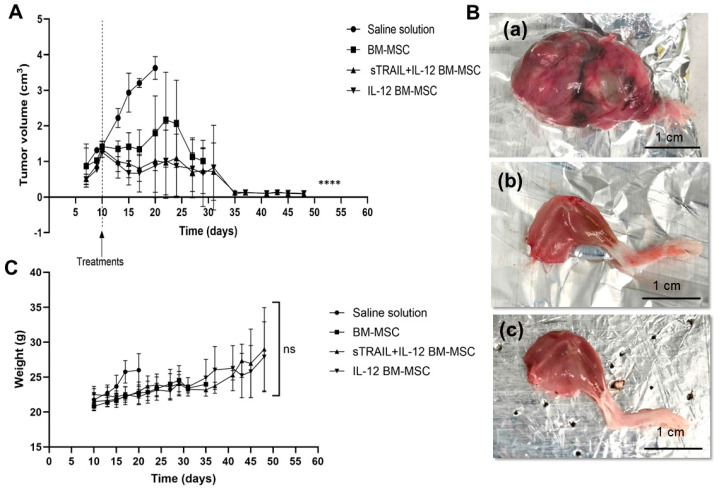
Application of BM-MSC expressing sTRAIL and IL−12 in syngeneic lymphoma murine model. (**A**) Treatments and tumor growth. Data represent media ± standard deviation. (**B**) Representative images of macroscopic analysis of the right lower leg of treated mice evaluated after sacrifice. (**a**) Tumor of mice from the control without treatment at day 20 post-inoculation of lymphoma cells. Right gastrocnemius of groups treated with (**b**) sTRAIL + IL−12 MSC and (**c**) IL−12 MSC at day 50 post-inoculation of lymphoma cells. (**C**) Mice weight during experiment. Media value ± standard deviation. BM- MSC, bone marrow mesenchymal stem cells; sTRAIL, soluble tumoral necrosis factor-related apoptosis-inducing ligand; IFNβ, beta interferon; IL−12, interleukin 12. ns = *p* > 0.05, **** *p* < 0.0001, n = 5.

L5178Y cells transduced cells can develop solid tumors when intramuscularly implanted. Solid tumors were induced in the right gastrocnemius of five groups of BALB/c mice, using a transduced lymphoma L5178Y cell line to express the reporter genes GFP/mCherry. The administration of BM-MSC overexpressing sTRAIL + IL-12 and IL-12 significantly reduced tumor growth compared to saline solution and naïve BM-MSC (*p* < 0.05, one-way ANOVA, Tukey’s multiple comparison). Interestingly, treatment of naïve BM-MSC at first reduces tumor growth, but by day 20, tumors continue their progression (Figure 5A). Additionally, there were no significant differences in mice’s weight related to treatment application (*p* > 0.05, one-way ANOVA). Results are shown in Figure 5C.

**Figure 6:**
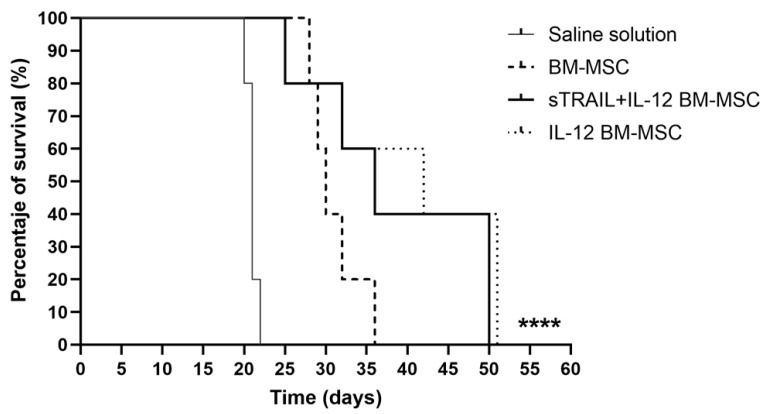
Survivor percentage of treated mice. BM-MSC, bone marrow mesenchymal stem cells; sTRAIL, soluble tumoral necrosis factor-related apoptosis-inducing ligand; IFNβ, beta interferon; IL-12, interleukin 12. n = 5 per group. **** *p* < 0.0001.

A significant difference in survival exists between MSC-expressing transgenes and the saline solution group (*p* < 0.0001). Moreover, survival was compared for each group (*p* < 0.05 Log-Rank [Mantel–Cox]). Experiments were carried out until day 50 (Figure 6).

The second control was the naïve BM-MSC group, in which all mice were sacrificed by day 36. The percentages of survival in the remaining groups were 60% sTRAIL + IL-12 BM-MSC and 60% IL-12 BM-MSC; however, there was no significant difference between these groups and naïve BM-MSC (*p* > 0.05, Log-Rank [Mantel–Cox]). These surviving mice were sacrificed by day 50; nevertheless, there was no solid tumor in the right gastrocnemius. 

A macroscopic analysis showed that the lower right leg of the mice was affected. The legs were isolated, weighed, and macroscopically described. An ovoid neoplasia covered by fibroconnective tissue with lumps and vasculature was observed in the negative control group (saline solution). In contrast, mice treated with transduced MSC overexpressed sTRAIL plus IL-12 or IL-12, which was only present in skeletal muscle tissue (Figure 5B).

### 3.5. IL-12 MSC and sTRAIL plus IL-12 MSC Reduce Tumor Cell Infiltration and Metastasis

**Figure 7:**
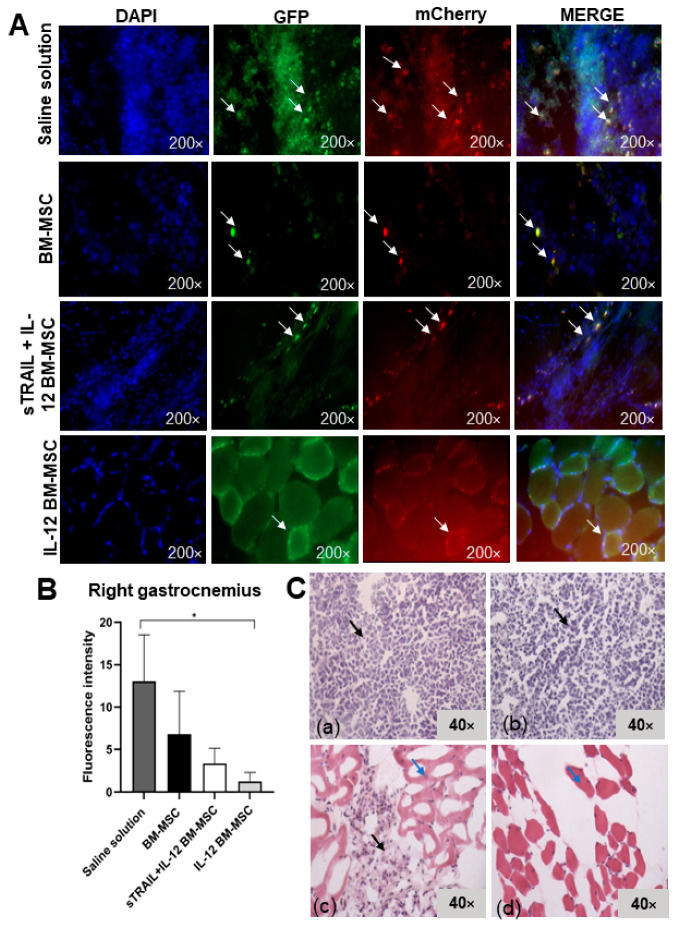
Histologic analysis of the right gastrocnemius. (**A**) Fluorescent cell signal in tissues from experimental groups: (**a**) saline solution, (**b**) naïve MSC, (**c**) sTRAIL + IL-12 MSC, (**d**) IL-12 MSC. Transduced L5178Y lymphoma cells marked with mCherry and GFP reporter genes in red and green. Cell nucleus stained with DAPI in blue. White arrows show a tumoral cell expressing reporter genes. (**B**) mCherry fluorescent signal. * *p* < 0.05. (**C**) Right gastrocnemius with hematoxylin–eosin stain: (**a**) saline solution, (**b**) naïve MSC, (**c**) sTRAIL + IL-12 MSC, (**d**) IL-12 MSC. Black arrows show tumor cell infiltrates; blue arrows show skeletal muscle cells. Eight random field analyses by three different observers. Bright field, 40×. BM-MSC, bone marrow mesenchymal stem cells; sTRAIL, soluble tumoral necrosis factor-related apoptosis-inducing ligand; IFNβ, beta interferon; IL-12, interleukin 12.

**Figure 8:**
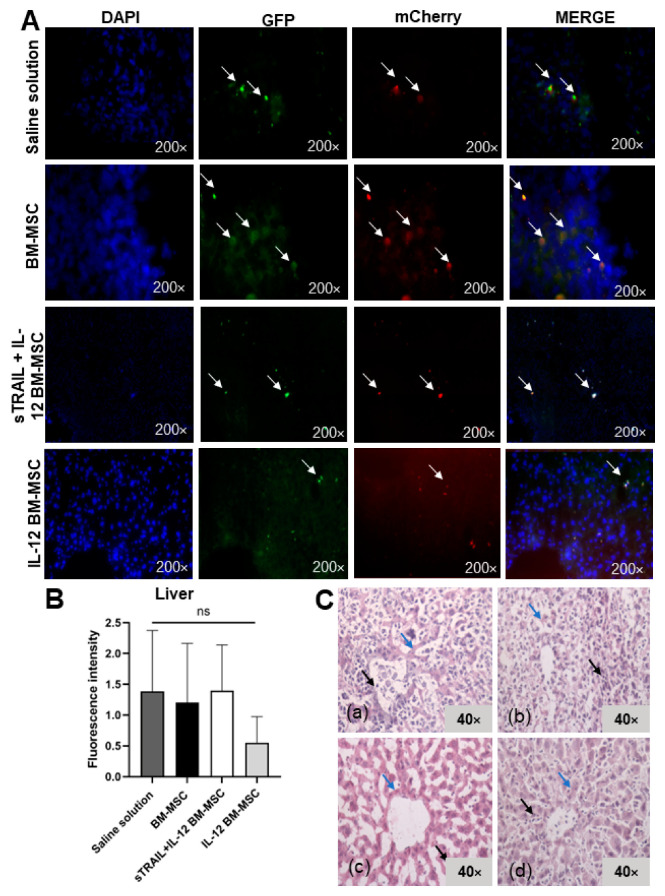
Histologic analysis of the liver. (**A**) Fluorescent cell signal in tissues from experimental groups: (**a**) saline solution, (**b**) naïve MSC, (**c**) sTRAIL + IL-12 MSC, and (**d**) IL-12 MSC. Transduced L5178Y lymphoma cells marked with mCherry and GFP reporter genes in red and green. Cell nucleus stained with DAPI in blue. White arrows show tumor cells expressing reporter genes. (**B**) mCherry fluorescent signal. ns: *p* > 0.05. (**C**) Liver with hematoxylin–eosin stain. (**a**) saline solution (**b**) naïve MSC, (**c**) sTRAIL + IL-12 MSC, (**d**) IL-12 MSC. Black arrows show tumor cell infiltrates; blue arrows show hepatocytes. Eight random field analyses by three different observers. Bright field, 40×. BM-MSC, bone marrow mesenchymal stem cells; sTRAIL, soluble tumoral necrosis factor-related apoptosis-inducing ligand; IFNβ, beta interferon; IL-12, interleukin 12.

Since metastasis was observed in the first experiment, the liver, tumor, or a right gastrocnemius biopsy was extracted. There were swollen lymphoid ganglia from the inflammatory process. By fluorescence microscopy, we visualized transduced tumoral cells in the right gastrocnemius and the liver. However, it was impossible to detect transduced BM-MSC. We observed a reduced fluorescent signal of tumoral cells in tissues treated with IL-12 and sTRAIL plus IL-12. The fluorescent signal intensity of mCherry was quantified with Image J software. An analysis of the right gastrocnemius showed significant differences in fluorescence intensity levels (*p* < 0.05, Kruskal–Wallis) between the groups; however, only the group treated with IL-12 MSC presented a significant reduction against the saline solution group (*p* < 0.05, Dunn’s multiple comparison). There was also a reduction of fluorescence in livers, but this was not significant between the different treatment groups (*p* > 0.05, one-way ANOVA; *p* > 0.05, Bonferroni’s multiple comparison). Macroscopically, there were no tumor residues. Figure 7 and Figure 8 summarize these results.

**Figure 9:**
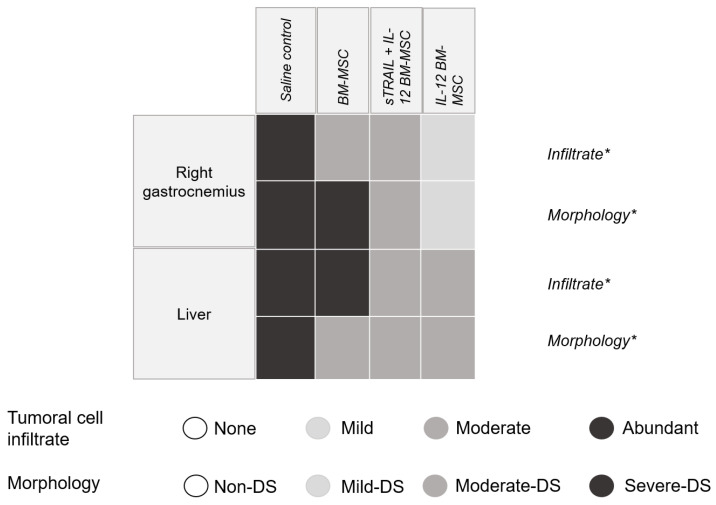
Histologic alteration, semiquantitative scale. The square includes the tumor/right gastrocnemius results and the liver histological analysis with hematoxylin–eosin stain. Changes in tumoral cell infiltrate and morphological alterations in different treatments are included. DS: dysplasia; tumoral cell infiltration: None 0%, Mild < 30%, Moderate < 60%, Abundant > 80%. Morphology: Non-DS, tissue without pathologic changes; Mild-DS, sinusoidal space increase; Moderate-DS, hepatocyte disintegration; Severe-DS, loss of hepatic parenchyma structure. * *p* < 0.001, chi-square.

Histologic alterations in the right gastrocnemius were analyzed by H&E stain. As previously mentioned, we established a semiquantitative scale of histologic alterations to classify the tumoral cell infiltration grade and morphology (Figure 9). The statistical analysis showed a significant difference between treatments (*p* < 0.0001, chi-square test) regarding tumoral cell infiltrates and tissue morphology in tumoral tissue samples of the right gastrocnemius and livers. The right gastrocnemius showed the best results with IL-12 BM-MSC treatment. It showed fewer morphologic alterations, more structured muscle tissue, and fewer tumoral cell infiltrates inside the tissue. In the liver, we observed a reduction of metastasis development since tumoral cells were reduced, and there was less tissue dysplasia. However, no treatment avoided metastasis progression. Thus, MSC treatments expressing IL-12 and sTRAIL reduce tumoral cells effectively when they are intratumorally applied in large tumors (1–2 cm^3^ tumors) and could reduce metastasis development. 

## 4. Discussion

BM-MSC has been widely explored as a platform for gene delivery in cell therapy and cancer. Studies have demonstrated that MSC can enter into tumor stroma and improve the release and distribution of therapeutic agents [14]. Allogenic and xenogeneic fibroblast cell lines have been used for neoplasia treatments in clinical trials; however, a recurrent problem is high immunogenicity, which produces a fast loss of transgene expression due to cell elimination [15].

We were able to take BM-MSC and develop full characteristics of MSC, such as plastic adherence, at least a 95% expression of CD105 and CD90 markers, and a lack of expression of hematopoietic markers such as CD34 (at least 2%). Moreover, the cells were able to differentiate into other lineages [16]. Lentiviral transduction was used to overexpress sTRAIL and IL-12 transgenes. In addition to the gene sequence, lentivirus included GFP as a fluorescent reporter. sTRAIL BM-MSC presented an efficiency of 88%, and IL-12 BM-MSC 77%, similar to that reported by other research groups [4,17]. In addition, Western blot showed protein bands characteristic of sTRAIL and IL-12. 

There are multiple studies of MSC expressing sTRAIL and IL-12 as proapoptotic and antitumoral strategies [4,18,19]; however, their combination has not been demonstrated. IL-12 regulates anti-tumor immunity, in a more precise manner IL-12 increases cell-mediated anti-tumor immunity. Briefly, IL-12 has been demonstrated to (i) enhance TH1 cell differentiation, (ii) increase T and NK cell activation and cytotoxicity, and (iii) inhibit or reprogram immunosuppressive cells such as tumor-associated macrophages (TAMs) and myeloid-derived suppressor cells (MDSCs). IL-12 further causes a substantial amount of IFNγ to be produced (a cytostatic/cytotoxic and anti-angiogenic agent), in addition, IL-12 can upregulate MHC I and II expression on tumor cells for improved antigen recognition and lysis. As a result, administration of IL-12 directly into the tumor may promote and activate tumor infiltration immune cells, which can eliminate tumor cells [20]. On the other hand, sTRAIL does not require cell-to-cell contact for apoptotic activity, moreover, sTRAIL can penetrate intratumorally even when the tissue is heavily fibrous or within a highly inflammatory microenvironment triggering apoptosis primarily in tumor cells [4]. The purpose of this study was to better understand the combining of sTRAIL and IL-12, with the idea that this relation could increase sTRAIL’s proapoptotic activity via boosting T-cell immune response (T-helper 1 and cytotoxic T-cell immunity). In addition, naturally occurring intratumor microenvironment TRAIL is also expressed by natural killer cells, T-cells, NK, T cells, dendritic cells, and macrophages [4,19,21] which helps potentiate the effect of sTRAIL expression of MSC. Thus, this combination could potentially be more efficient in overall eliminating tumoral cells as there is an increase in cell immunity, which is frequently attenuated in tumors by immunotolerance induction orchestrated by the tumor microenvironment [22].

We found that both BM-MSC treatments expressing sTRAIL and IL-12 or only IL-12 significantly reduced tumor growth (*p* < 0.05) and showed less tumor weight (*p* < 0.05) without short-term secondary effects [4]. Moreover, these treatments increased the overall survival of mice (*p* < 0.0001). In a previous study [6], individual use of IFN-β BM-MSC and sTRAIL BM-MSC allowed the survival of 25 and 50% of experimental groups until day 42; nevertheless, this combination generated an additive effect by increasing the percentage of surviving mice to 62.5%. In this study, 60% of mice survived until day 50 with IL-12 BM-MSC. The combination of sTRAIL and IL-12 achieved the same survivability of 60%. Thus, there was not an additive effect. Although we detected levels of sTRAIL in pg/mL magnitudes by BM-MSC that according to other authors these levels can induce apoptosis in tumor cells, the effect of IL-12 was even better as it activates the immune response against tumoral cells, as mentioned before [4,19,21]. We consider the lack of quantification of IL-12 levels in BM-MSC and both (sTRAIL and IL-12) directly in the tumor mass as a limitation of the study. 

Malignant ascites showed that 80% of mice treated with IL-12 MSC survive longer than naïve MSC, persisting until day 40 [23]. In gliomas, 70% of mice treated with IL-12 BM-MSC survived more than 90 days [21], whereas TRAIL MSC-treated mice survived until day 60 [24]. Lung metastasis in a murine model showed that mice treated with IFN β MSC had an average survival of 60 days [25]. Therefore, IL-12 MSC is proposed as a better strategy for in vivo cancer models such as glioma, renal cell carcinoma, breast, melanoma, and hepatoma [15,19,21,26,27]. However, combining IL-12 with other agents to potentiate cancer cell death by other pathways besides immunotherapy should be explored. 

Regarding tumoral cells, the GFP/mCherry L5178Y cell line presented high expression of the DR5 TRAIL receptor, indicating its possible sensibility to cell death with this treatment. L5178Y has not been reported as capable of developing metastasis; however, in our results in a murine model, we detected that the GFP/mCherry L5178Y cell line could migrate to the liver (in 22 days). Furthermore, we confirmed the morphological characteristics associated with setting up metastatic disease, offering an in vivo model to study antineoplastic drugs or agents with antitumor and antimetastatic activity. A macroscopic analysis of treated right gastrocnemius allowed a comparison of anatomopathological differences, suggesting that in contrast to the saline solution control group, where the presence of neoplastic tissue was evident, in transduced BM-MSC groups, only striated skeletal muscle was observed in the right lower leg. mCherry fluorescence intensity showed that the IL-12 BM-MSC treated group had a significantly reduced (*p* < 0.05) fluorescent signal in the right gastrocnemius. It is important to note that other studies required multiple doses of MSC to observe the antitumoral [4,18] and antimetastatic activity [21]. In our study, the treatments showed a reduction in tumor growth in large-volume tumors, and metastasis was reduced but not eliminated. The poor antimetastatic effectivity is related to the lack of homing to the liver due to its limitation in tumor stroma and cell death [4]. Thus, changes in treatment with the application of multiple doses of MSC should improve the outcome, as demonstrated at the study’s end when transduced BM-MSC were eliminated from the inoculation site.

Using a semiquantitative scale, we found that IL-12 BM-MSC treatment has the greatest efficiency for reducing morphological alterations and tumor cell infiltration in the right gastrocnemius. Combined treatment with sTRAIL plus IL-12 or IL-12 alone similarly reduced the tumor cell infiltrate and morphological alterations related to metastases in the liver. Although IL-12 had an antimetastatic effect in other studies [15,21,23], in this study, tissue changes related to metastasis continued in the treated groups. This finding could be related to MSC doses and the inoculation site. Thus, BM-MSC overexpressing antitumoral products can limit tumor growth and improve apoptosis when focally administered [28]; nevertheless, they can reduce metastasis development.

TRAIL induces apoptosis by activation of the caspase 8 receptor [4]. However, even with proapoptotic receptors, tumoral cells can develop resistance to apoptosis, as demonstrated in colon, breast, and liver cancer and leukemia. The main mechanism is the overexpression of FLIP, which in turn blocks the DISC complex. Another mechanism is the overexpression of BCL-2 anti-apoptotic members, which inhibit apoptosis mediated by mitochondria. Moreover, TRAIL-resistant cells possess higher activation of the NF-kβ pathway related to cell proliferation [29]. The lack of additive effect of TRAIL and IL-12 could be associated with resistance development of lymphoma cells, which keeps as a perspective for future research. 

There are some concerns about the systemic use of IL-12, as there is a risk of developing metabolic acidosis, hepatitis, renal failure, and death in clinical trials. Furthermore, local IL-12 application does not generate toxicity and can eliminate tumors, as it is more regulated by the organism [17,19]. IL-12 induces IFN-γ release and infiltration of CD4+, CD8+ T cells, NK cells, and dendritic cells into the tumor, evidencing its potential use in immunotherapy [23,30]. At the same time, MSC gene modification with adenoviral vectors for IL-12 expression could present more intense antitumoral effects than recombinant plasmid vectors or naïve MSC. Moreover, even when it is demonstrated that naïve MSC can produce limited amounts of TRAIL [31], the use of naïve MSC might present adverse effects as it increases cancer cell migration due to its immunosuppressor properties in hypoxia [14]. We observed that mice tumors treated with naïve BM-MSC resume growth after day 20; further, they present abundant tumoral cell infiltrates and severe dysplasia in the right gastrocnemius and the liver. Current research focuses on using MSC, or adipose stem cells (ASC) stimulated with cells such as M1 macrophages to improve the production of TRAIL without gene manipulation for short-term clinical application [32]; however, this concept needs more research. 

This study confirms the antitumoral activity of IL-12 BM-MSC as an alternative treatment for lymphoma. Although we anticipated that the combination of treatments potentiated the action of both proteins, the in vivo model showed the same efficacy as the individual use of IL-12. This finding indicates a possible resistance mechanism of cancer cells to apoptosis by TRAIL or the need for another delivery option for treatment. Nevertheless, our results bring new insights and questions for cancer research. 

## 5. Conclusions

We found that BM-MSC expressing sTRAIL plus IL-12 or only IL-12 is a potential strategy against lymphoma solid tumors, reducing tumor growth and increasing survival in a murine lymphoma model with only one dose of 1 × 10^6^ cells. However, for this model, there was not a significative additive effect with BM-MSC expressing sTRAIL. Moreover, both treatments reduced the progression of morphological changes related to liver metastasis.

## Data Availability

The data supporting these findings can be found at the School of Medicine of the Autonomous University of Nuevo Leon, Biochemistry and Molecular Medicine Department.
